# Long-term Distribution, Nesting Activity, Hatching Success and Environmental Influences of the Hawksbill Turtle in Peninsular Malaysia. Part 1: A Case Study from 2015–2019

**DOI:** 10.21315/tlsr2026.37.1.1

**Published:** 2026-03-31

**Authors:** Sarahaizad Mohd Salleh, Shahrul Anuar Mohd Sah, Saufi Affandi Talib, Siti Mastura Daud, Azizah Ibrahim

**Affiliations:** 1 Environmental Science Center, H10 Research Complex, Qatar University, 2713 – Doha, Qatar; 2 School of Biological Sciences, Universiti Sains Malaysia, 11800 USM Pulau Pinang, Malaysia; 3 Melaka State Fisheries Office, 75350, Batu Berendam, Melaka, Malaysia

**Keywords:** Distribution, Environmental, Hawksbill Turtle, Nesting, Peak Season, Taburan, Persekitaran, Penyu Karah, Sarang, Musim Kemuncak

## Abstract

Hawksbills’ (*Eretmochelys imbricata*) status in the state of Melaka is continuously being assessed due to Melaka being the only prominent location for hawksbills in Peninsular Malaysia. Over five years of nesting season, results of the survey revealed the cumulative nests collected the most were in Padang Kemunting and Kem Terendak with a range between 400 nests–700 nests yearly. A year-round nesting trend was observed, and the preferable peak period tends to occur between June to August almost every year. The clutch size ranged from 5 to 243 eggs over five years of the survey. An increase in temperature and rainfall amount will influence the increasing number of nests, while humidity and precipitation did not impact the nesting per month. This article presents updated nesting statistics for hawksbill turtles, showing an increase in annual nesting activity over the five-year period from 2015 to 2019 in Melaka, compared to the earlier years of 2013–2014. A greater comprehension of hawksbills’ reproductive potentiality under environmental force will aid conservation and management aims.

## Abstrak

Status penyu karah (Eretmochelys imbricata) di Melaka dikaji secara berterusan disebabkan Melaka adalah satu-satunya tempat penting yang dikenali sebagai lokasi utama pendaratan penyu karah di Semenanjung Malaysia. Sepanjang kajian yang dijalankan selama lima tahun (2015–2019), keputusan kajian menunjukkan tempat tumpuan utama taburan penyu bersarang adalah di Padang Kemunting dan Kem Terendak, dan berjulat diantara 400–700 sarang pada setiap tahun. Turun-naik jumlah kutipan sarang tahunan dipantau dan diketahui musim kemuncak terarah berlaku pada Jun hingga Ogos pada setiap tahun. Saiz sarang pula berjulat daripada 5 hingga 243 biji telur menerusi tinjauan dalam tempoh lima tahun. Peningkatan suhu persekitaran dan jumlah taburan hujan memberi kesan kepada peningkatan jumlah sarang yang dijumpai pada setiap bulan, manakala kelembapan dan kepadatan hujan pula tidak mempengaruhi jumlah sarang yang dijumpai. Kajian ini bertujuan mengemas kini data terbaharu statistik penyu karah, berlakunya peningkatan jumlah sarang tahunan dalam tempoh lima tahun di Melaka berbanding pada tahun-tahun sebelumnya (2013–2014). Pemahaman yang lebih besar tentang potensi pembiakan penyu karah dan hubung kaitnya dengan pengaruh alam sekitar akan membantu ke arah tumpuan pemuliharaan dan pengurusan.

**HIGHLIGHTS**

- The updated statistics of hawksbill nesting are between 300–600 nests per year between 2015–2019.
- Latest nesting status in Melaka has shown a steady increase.
- There was a significant effect of air temperature and rainfall on nesting density, which shows the environmental influence on nesting behaviours.

## INTRODUCTION

Hawksbill can be found in clear water estuaries and mangroves (Murphy *et al*. 2018; Beggs *et al*. 2007). Their wide distribution includes the Caribbean Sea and the Atlantic Ocean (Murphy *et al*. 2018), and the Pacific and Indian Oceans (Chan *et al*. 1999; Limpus 2009; Stiles 2009). Habitat loss (Balazs 1976), overexploitation, tortoiseshell trade (Sáenz-Arroyo *et al*. 2006), conflict with humans, direct hunting for meat and commercialisation are major threats to hawksbill turtles in many parts of the world. This species has been widely hunted because of the beautifully marked scutes (Burnie & Wilson 2001). Historically, the high demand for the hawksbill turtle’s richly patterned scutes has led to significant population declines due to intensive harvesting (Groombridge & Luxmoore 1989). The first documented surveys of wildlife markets in Vietnam, conducted between 1990 and 1991, reported widespread sale of tortoiseshell in Ho Chi Minh City and Vung Tau (Stiles 2009). China and Japan have prominently featured in this trade; described as hawksbill shells coveted in the luxury handmade arts and crafts markets, simultaneously, this was also popular in South Korea and Taiwan (Lam *et al*. 2012). For example, between 1970 and 1988, shells from approximately 272,700 hawksbill turtles was imported into Japan (Bjorndal *et al*. 1993). Although Vietnam’s involvement in the trade continued illegally into the 1990s, it declined significantly after Japan withdrew its reservation to the CITES listing in 1994, effectively ending legal commercial trade (Stiles 2009). In addition, the main threats to hawksbill conservation are anthropogenic impact factors, such as degradation of the nesting area, pollution and fisheries bycatch (da Silva *et al*. 2015). The hawksbill turtle is a critically endangered species in tropical and subtropical waters around the world (Mortimer & Donnelly 2008). Current estimates indicate a 90% decline in the worldwide population of hawksbill turtles in all major oceans over the last 100 years (Mortimer & Donnelly 2008; IUCN 2025).

Many biological and physical variables, including anthropogenic perturbations (artificial light pollution, human presence and houses on nesting beaches) (Kikukawa *et al*. 2001; Pike 2009), influence nest-site selection by sea turtles. Relevant variables include beach morphology (orientation, slope and width), a series of small-scale factors, such as sand granulometry and nest characteristics (humidity, pH and temperature), and nearshore oceanographic conditions (Horrocks & Scott 1991; Ficetola 2007; Kelly *et al*. 2017).

There are many potential impacts of climate change on sea turtles, including a decline in hatching success with incubation temperatures reaching lethal limits and loss of nesting habitat due to a rise in sea level (Katselidis *et al*. 2012). Water temperature also affects inter-nesting intervals of sea turtles (Hays *et al*. 2002) and air temperature and precipitation in nesting beach habitats affect sex ratios of hatchlings and survival of eggs (Santidrián *et al*. 2012; Lolavar & Wyneken 2015). Additionally, the influence of sea surface temperature also causes shifts in the nesting patterns of sea turtles have already been illustrated (Mazaris *et al*. 2009). Therefore, Patel *et al*. (2016) suggested that sea turtles are exposed to environmental change, which affects both their terrestrial (nesting beaches) and oceanic habitats.

For the stewardship of ecosystems and biodiversity, evaluating the potential impacts of climate change on individual species and populations is essential (Saba *et al*. 2012). A greater understanding of hawksbills’ reproductive capabilities under environmental pressure will aid conservation and management attempts (Chatting *et al*. 2018). Global variations in nesting ecology have been shown to drive regional fertility (Mortimer & Bresson 1999). Variation in nesting ecology includes variation in clutch frequency, clutch size, nesting season durations, remigration intervals and morphological size of nesting females (Chatting *et al*. 2018) and this variation influences regional differences in nesting dynamics (Mortimer & Bresson 1999).

The objectives of this study were to identify the sum spatiotemporal of hawksbill nesting and its variance (mean and median) at Melaka, Peninsular Malaysia across a five-year, as well as to record the dynamics of beach coverage by hawksbill nesting activity during a given period of time. This study aimed to test the hypothesis that hawksbill nesting ecology such as clutch size and nesting distribution experiences the greatest influence from environmental factors (i.e., air temperature, humidity, precipitation and rainfall) from the 2015–2019 nesting periods. We also would like to formally document the findings of a five-year monitoring project on hawksbill nesting in Melaka and compare it to other nesting densities across various locations global. Yearly nesting data of hawksbill from 2015 to 2019 were retrieved from the Department of Fisheries (DoF) with their permission and approval before the research outcome is officially published for readers. In Malaysia, hawksbill has been recognised to be mainly in Melaka (Mortimer *et al*. 1993), Sabah (Chan *et al*. 1999) as well as distributed in Terengganu (Chan 2010; 2013) and Negeri Sembilan (Mortimer *et al*. 1993). In Melaka, the earliest documentation of hawksbill was recorded by Mortimer *et al*. (1993) and was further updated after 21 years by Sarahaizad *et al*. (2018). The observation was conducted in 2013 and 2014, which shows slightly increasing numbers of hawksbill population in Melaka after > 20 years (Sarahaizad *et al*. 2018). This research provides the latest view on geographic nesting distribution, seasonality and nesting density per beach over five years in Melaka. Emergence hour and microhabitat preferences were also analysed as the data were collected by DoF for two years in 2017 and 2018. The DoF provided two years of data (2017–2018) for emergence hours, microhabitat and nest site preferences due to the lack of staff to record data in the previous years. This article is important as it highlights the status of hawksbill in Melaka, which is crucial for conservation.

## MATERIALS AND METHODS

### Study Area

**FIGURE 1:**
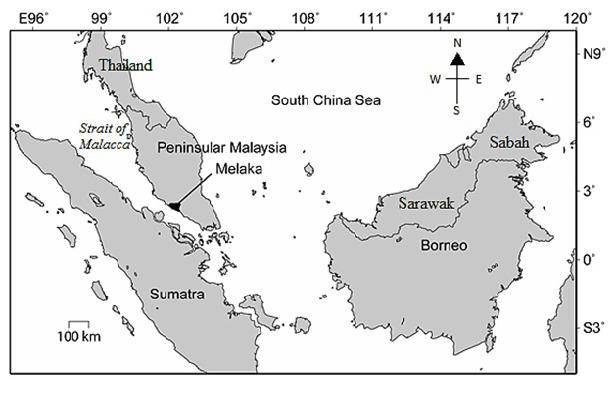
Map of research site located on the west coast of Peninsular Malaysia.

Sampling and data collection were performed on nesting beaches of Melaka (Fig. 1) by DoF’s staff, together with Padang Kemunting Turtle Conservation Centre’s staff, hired egg collectors and World Wildlife Fund’s (WWF) team. Currently, DoF has hired 13 patrollers to monitor the nesting beaches. All beaches were consistently monitored for 365 days from January 2015 to December 2019 (five years). Nocturnal beach surveys were performed according to the standard operating procedures by DoF (Sukarno *et al*. 2007; Rusli *et al*. 2020) to avoid disturbance for hawksbill as we surveyed more than one beach per night. For example, all patrollers provided full co-operation in instances such as communicating using a walkie-talkie to avoid miscommunication during the intensive beach survey. Patrollers walked slowly along the sandy beach using minimal light to verify any landing tracks. To avoid disturbances, nesting data were recorded and relocated after the turtle had finished nesting and begin returning to the sea. Camera flash was disallowed during the nesting process. During the survey, the patroller and researcher were advised to wear properly covered shoes, socks and dark-coloured shirts and trousers. No slippers and short pants were allowed as there is thorny vegetation at every nesting beach. Nests encountered on the beach were placed in Styrofoam boxes and transported slowly by car directly to the hatchery at the Padang Kemunting Turtle Conservation Centre. The eggs were then re-buried in the hatchery (nest depth is between 60 cm–70 cm), and the entire process had to be done within five hours of being laid by the mother turtle. Nesting output data that were consistently recorded include a daily number of nests per beach and clutch size per nest. Nesting emergence hours were also recorded from the time turtle landed on the beach from the tidal line.

A beach survey was performed from 20:00 h to 06:00 h every night at six nesting beaches by placing between one to three patrollers at one time according to the beach length. High density nesting beaches include Padang Kemunting, Kem Terendak, Meriam Patah, Tanjung Dahan, Tanjung Serai and Pengkalan Balak. Consistent surveys were performed by patrollers every night for 365 days, as these beaches hold the highest nesting density of hawksbill turtles in Melaka based on a previous publication by Sarahaizad *et al*. (2018). Meanwhile, nesting surveys at Balik Batu, Pasir Gembur, Tanjong Bidara, Teluk Belanga, Sungai Kertah, Teluk Gong, Kuala Linggi, Sungai Tuang and Kampung Teluk were dependent on local eggs collector, and DoF are buying eggs from them at a capped price of RM1.15 per egg, and these eggs were incubated at the hatchery. Due to the good relationship between the locals and DoF staff, nests encountered in remote areas were easily sold to the DoF staff to help reduce poaching. Lastly, sampling at Sungai Udang, Kampung Tengah, Tanjung Kling, Kelebang, Puteri Beach and other nesting beaches were dependent on reports as these beaches hold the least nesting.

In the meantime, WWF teams assisted in monitoring certain beaches, particularly the high-density ones mentioned above. In Pulau Upeh, the DoF management allowed the WWF team to take full responsibility for the patrolling schedule and egg collection. Pulau Upeh itself is an island that is under the management of the Melaka State Government. Consequently, the survey at this island is a bit costly as it needs boat transportation between the mainland and the island, and the boat schedule to the island depends on the tidal level.

### Nest Site Preferences and Microhabitat

The microhabitat preferences of the hawksbill turtles were investigated to identify leading factors of nesting site selection from 1 April 2017 until 31 October 2018, along the beaches of the State of Melaka, on the west coast of Peninsular Malaysia. The nest locations were measured using a 50.0 m measuring tape (± 0.1 cm) and the distance from the nest to the highest spring tide line was recorded. The distance from the highest spring tide line, interpreted as the distance from the nest to the marked spring tide, is taken as the line of marine detritus on the beach (Santos *et al*. 2016). The habitat preferences of the hawksbill turtles were categorised as follows:

1. Vegetation and grassy areas.
2. Vegetation areas.
3. Open sand.
4. Grassy area.

Vegetation areas were designated as areas with shrubs, woody and bushy vegetation. Grassy areas were specified as area covered only with grasses; while the vegetation and grassy area were areas composed of both elements. Open sand areas had totally no vegetation coverage.

### Environmental Data

Mean monthly environmental factors data (temperature, rainfall, rain days, humidity and precipitation) from January until December of 2015–2019 (60 months) were retrieved from the Malaysian Meteorological Department at Batu Berendam, Melaka station (Latitude: 2°16′ N and Longitude: 102°15′ E, Elevation: 8.5 m).

### Statistical Analysis

All results were analysed using SPSS 17.0 version, GPS visualiser (GPS Visualiser 2015), and Microsoft Excel.

## RESULTS

A total of 2,522 nests were recorded for five years of observations in Melaka, Malaysia between 2015 and 2019 (ranged = 350 nests–600 nests, mean ± SD = 504.4 ± 87.12). In 2015, a total of 418 nests were observed and 18 nesting beaches were identified (mean nests per beach ± SD = 19.90 ± 24.79, median = 8.00) (Table 1). However, the number of nests reduced in 2016, as there were only 383 recorded nests and 14 nesting beaches (mean nests per beach ± SD = 18.24 ± 29.57, median = 3.00). The number of nests shows a trend of increasing in 2017 onwards. A total of 573 nests were collected in 2017 and 13 nesting beaches were identified (mean nests per beach ± SD = 27.29 ± 43.85, median = 7.00). Furthermore, there are 600 nests collected in 2018 and this is the highest annual nesting record within five-years studies (mean nests per beach ± SD = 28.57 ± 42.39, median = 9.00, *N* = 15 beaches). Lastly, a sum of 548 nests was recorded in 2019 with 13 identified nesting beaches (mean nests per beach ± SD = 26.10 ± 38.81, median = 6.00, *N* = 13 beaches).

**FIGURE 2:**
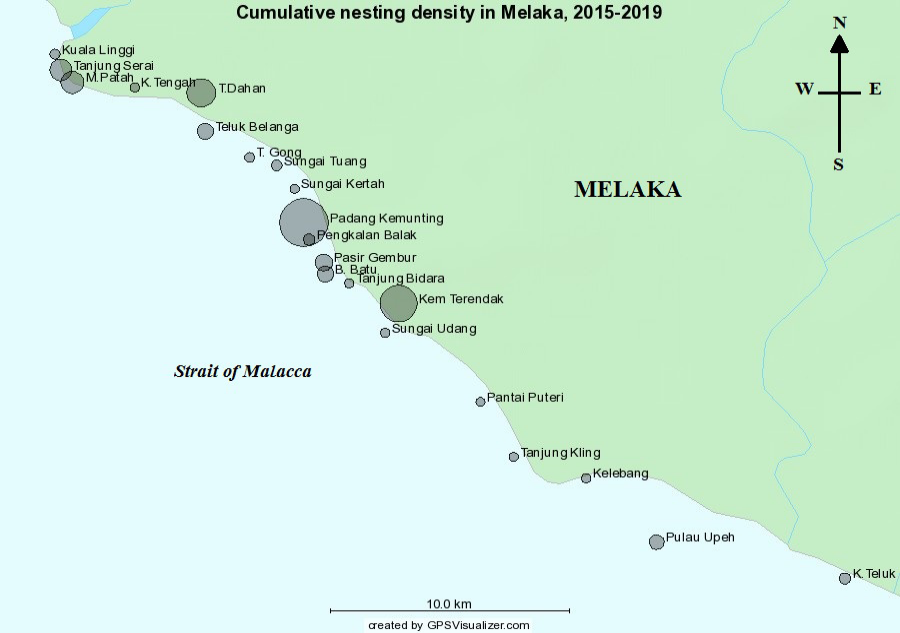
Nesting distribution of hawksbill over five years in Melaka nesting beaches, larger circle indicates larger density of nesting.

There are 21 nesting beaches identified within five-years and the beaches were plotted along the seashore of Malacca (Fig. 2). Most nests were collected at Padang Kemunting and follow by Kem Terendak with cumulative of 666 and 466 nests from 2015 to 2019 (Table 1). This was followed by Tanjong Dahan, Meriam Patah and Tanjung Serai with a cumulative of 330, 225 and 213 nests, respectively. Additionally, Pasir Gembur, Teluk Belanga, Balik Batu and Pulau Upeh has a cumulative nesting between 100 nests–150 nests. Meanwhile, Pengkalan Balak, Kampung Teluk, Sungai Tuang, Kuala Linggi and Teluk Gong recorded cumulative nesting between 10–99 nests. Lastly, least nesting were collected at Tanjung Bidara, Sungai Udang, Sungai Kertah, Kampung Tengah and Puteri Beach with cumulative nesting of < 10 nests. Previously, a sum of five nests were recorded in 2013–2014 at Kelebang and Tanjung Kling (Sarahaizad *et al*. 2018), however, no nest was recorded at these two beaches within 2015–2019. In conclusion, 21 nesting beaches were identified as hawksbill rookeries in Melaka where hawksbill turtles have historically nested since the research began in 2013–2014. These sites are monitored to track nesting activity, population trends and conservation success. However, between 2015 and 2019, nesting activity was recorded at only 19 of these beaches, indicating a decline (Table 1).

According to Table 1, a stable decline was observed for hawksbill nesting at Padang Kemunting, Meriam Patah and Tanjung Serai through the five years of observation. While, improvement can be seen at Kem Terendak and Tanjung Dahan due to increasing trend of nesting within five years at these beaches. The drastic reduction occurred at Teluk Belanga, which only six nests recorded in 2019, as compared to between 19–45 nests collected from 2015 until 2018. The clutch size ranged from 5 to 243 eggs in 2015 (mean ± SD = 122.86 ± 27.88, *N* = 331), 5 to 206 eggs in 2016 (mean ± SD = 71.31 ± 52.92, *N* = 383 nests), 5 to 199 eggs in 2017 (mean ± SD = 119.85 ± 11.51, *N* = 374 nests), 2 to 209 eggs in 2018 (mean ± SD = 87.99 ± 56.98, *N* = 600 nests), and 6 to 211 eggs in 2019 (mean ± SD = 79.19 ± 63. 33, *N* = 547 nests).

**FIGURE 3:**
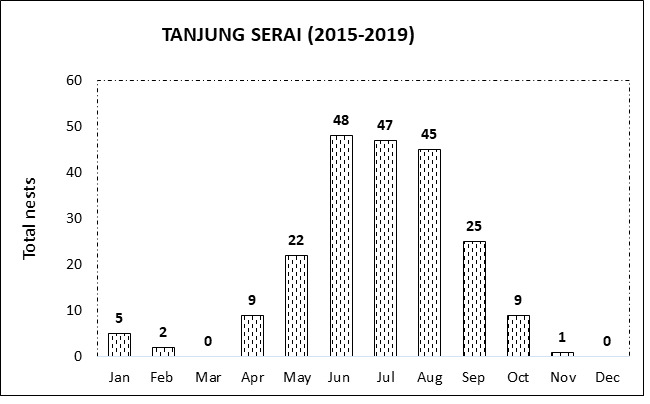

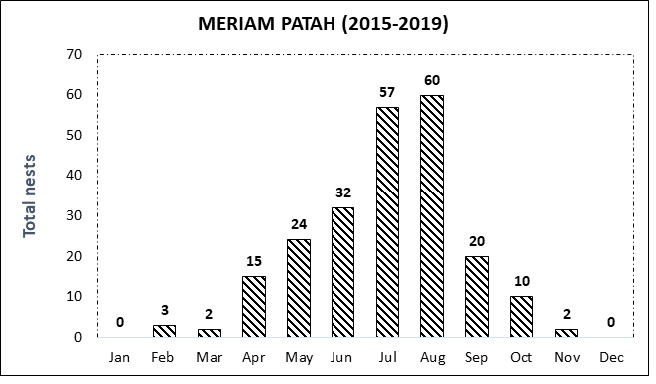

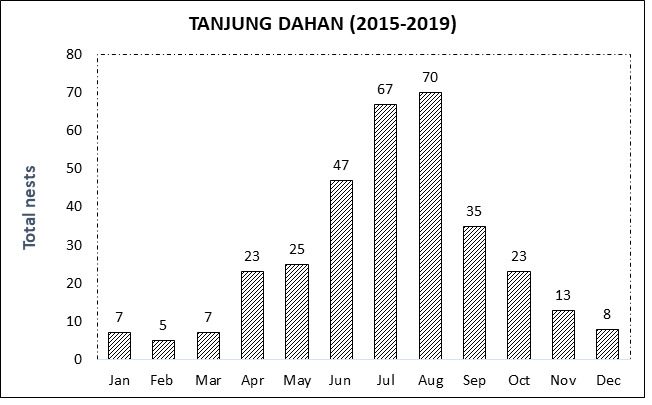

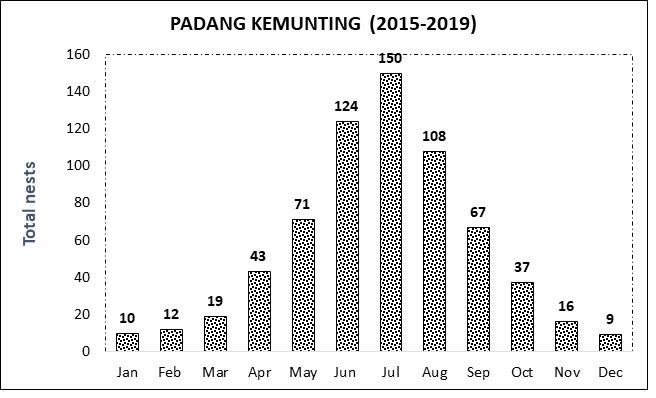

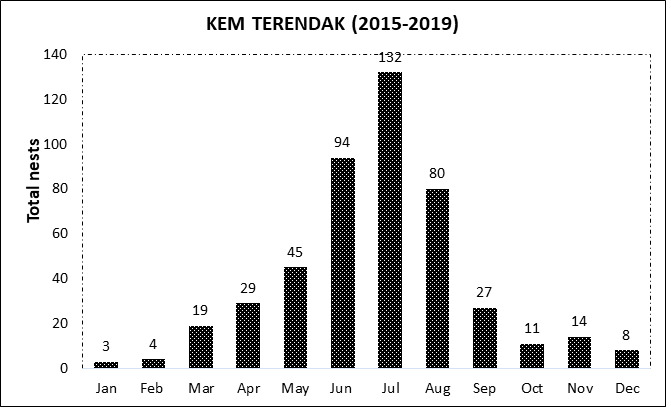
Nesting trend per months (cumulative from 2015–2019) for five important nesting beaches in Melaka. (a) Kem Terendak, (b) Padang Kemunting, (c) Tanjung Dahan, (d) Meriam Patah and (e) Tanjung Serai.

**FIGURE 4:**
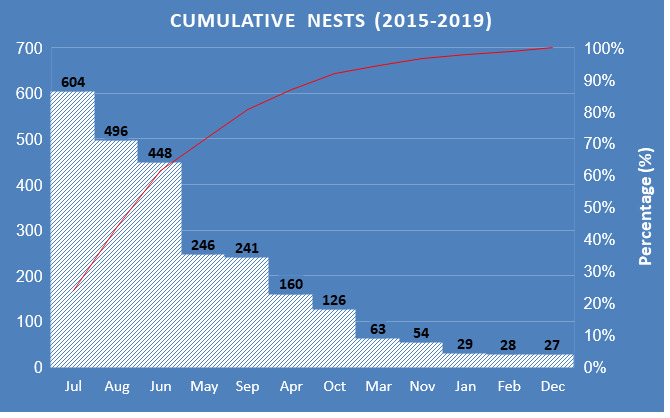
Cumulative nesting according to months for all the 21 nesting beaches in Melaka from 2015–2019. Graph indicates July, August and June is the peak time of nesting for turtle to nests. The inclined red line indicates the cumulative number of nests in percentage (%).

As shown in Figs. 3(a)–(e), year-round nesting was observed at almost all important nesting beaches, except in Tanjung Serai, where no nests were recorded in March and December within five years. Furthermore, the preferred peak period for hawksbill tends to occur between June, July and August almost every year which ranges between 32 to 150 per month (Fig. 3[a]–[e]) and the result within the survey time is summarised in Fig. 4 which gives an overall sum of 604, 496 and 448 nesting in July, August and June, respectively. After entering the peak of the season, the trend of nesting starts to decrease from September to February annually, also known as the low season, and when entering March, this marks the beginning of the season.

**FIGURE 5:**
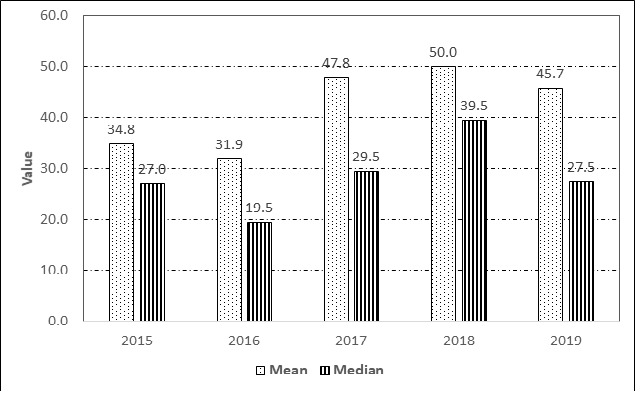
Sum of mean and median of nesting per year.

In addition, the total number of nests recorded from June to August has been summarised annually: 248 nests (59.33%) in 2015, 232 nests (60.57%) in 2016, 370 nests (64.57%) in 2017, 356 nests (59.33%) in 2018, and 342 nests (62.41%) in 2019. There was no statistically significant association between months and years, indicating that the seasonal nesting trend across the 12 months remained relatively consistent over the five-year observation period, Pearson’s correlation coefficient was *r* (60) = 0.115, *p* > 0.05, *N* = 60. The mean of nesting per year ranged from 31.9–50.0, and the median ranged from 19.5–39.5 (Fig. 5). Hawksbill nested a large number of clutch sizes of 212 eggs in 2013 and 243 eggs in 2015 (Sarahaizad *et al*. 2018). During the surveyed period, the largest clutch size was 201 eggs in 2015, 197 eggs in 2016, 199 eggs in 2017, 209 eggs in 2018 and 211 eggs in 2019.

**FIGURE 6:**
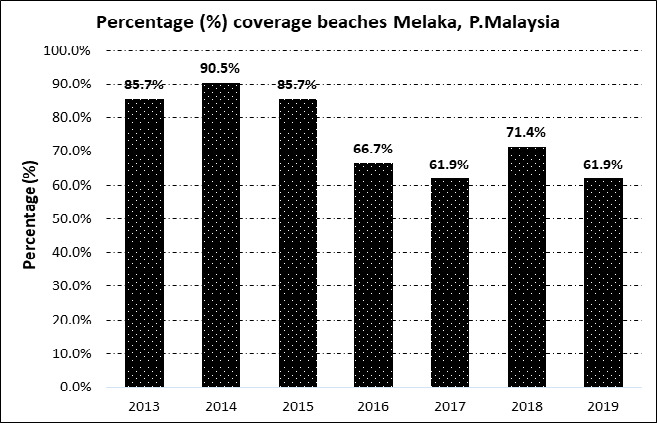
Percentage of beach coverage (%) per year in Melaka, Peninsular Malaysia.

Since 2013, the researchers have been identifying a total of 21 recorded beaches where hawksbills laid eggs. Although the nesting status in Melaka has shown a steady increase over the past seven years, a noticeable decrease in beach coverage percentage was observed from 2015 to 2019, with the exception of 2018 (Fig. 6). Previously, beach coverage reached the maximum of 90.5% where turtles landed at almost all beaches in Melaka in 2014.

Ex-situ methods were performed in Melaka due to anthropogenic perturbations. Eggs from the nests were incubated at the shaded hatchery and hatching success ranged from 38% to 61% yearly (Table 2). It has been observed that the short beach length from the water’s edge to the hatchery is less than 20 m. Furthering the study on beach characteristics and environmental conditions will contribute to valuable outcomes regarding the relationship with hatching success. Melaka is well known as a tourist spot for social beach activities (by the beach restaurant, homestay), and dense in human presence especially during the school holiday. This uncontrollable situation causes some nests to either be partially or fully poached (Table 3). The number of poached nests reduced by 27.9%, which is from 104 recorded in 2017 to only 75 poached nests recorded in 2018.

**FIGURE 7:**
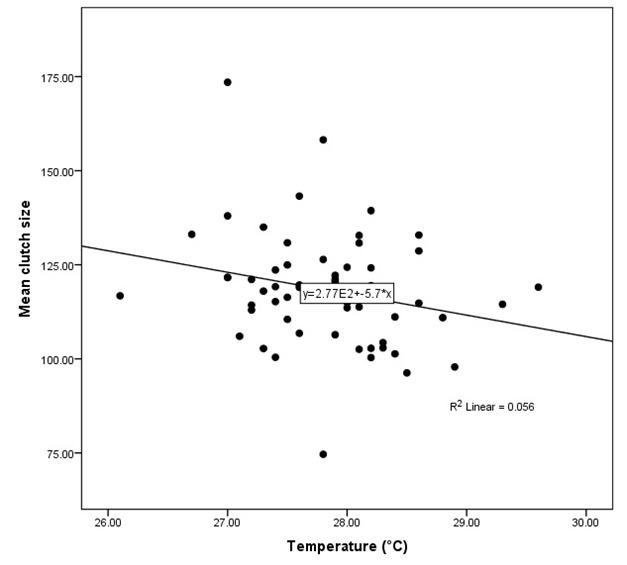
Linear regression analysis between mean clutch size and temperature.

A multiple regression analysis was performed to predict the distribution of nesting and the relationship with the environmental factors (temperature, humidity, precipitation and rainfall amount). Temperature and rainfall amount variables added statistically significance to the prediction that an increase in temperature and rainfall amount will influence the increasing number of nests, *p* < 0.05, while, humidity and precipitation did not impact the nesting per month (Table 4). Over five years, environmental factors influenced the number of clutch sizes laid during the oviposition process. Temperature variables added statistical significance to the prediction, *p* < 0.05 assignable to higher temperature resulted in fewer clutch sizes, even though the relationship is weak (Fig. 7). Conversely, humidity, precipitation and rainfall amounts did not affect the mean clutch size per month.

**FIGURE 8:**
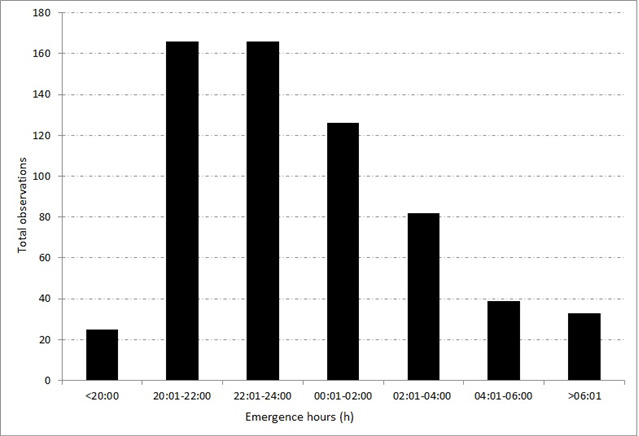
The sum of two-years observation on emergence hours in Melaka, Peninsular Malaysia.

The emergence hour during night-time was observed for 604 of a total of 637 nests for two years (2017–2018) (see Fig. 8). During nocturnal surveys, hawksbill emerges most frequently between 20:00–22:00 and 22:01–24:00 with equal 166 observations at both intervals. Diurnal nesting was also observed occurring from 06:01 with 33 observations. There is also a group of hawksbill turtles that prefer to land during sunset, after 8:00 PM, accounting for 3.92% of observations (25 females). Each of these turtles bore a different tag number on their flippers. The pattern of emergence hours was not equally distributed, χ^2^ = 495.075, *df =* 6, *p* > 0.05.

**FIGURE 9:**
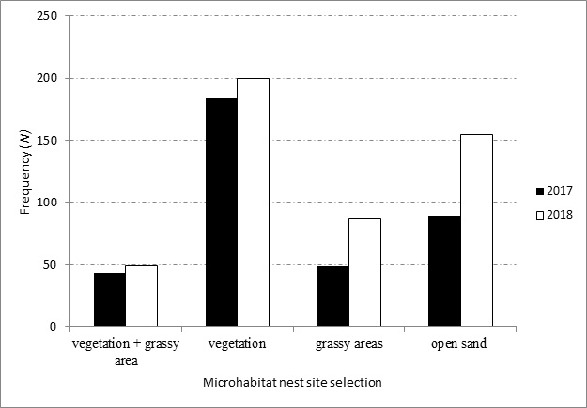
Microhabitat nest site selection in two-years (2017 and 2018).

Hawksbills demonstrated a preference for nesting in the vegetation zone with 50.3% of nests deposited here over the two years (184 observations in 2017 and 200 in 2018 (Fig. 9). Hawksbill also laid nests at open sand (31.9%) and within grassy areas (17.8%). The least habitat preference was within vegetation + grassy zone with 12.0% of observations. There is a statistically significant relationship between microhabitat preferences and years, [(χ^2^ = 0.000, *df* = 4, *P* > 0.001, *N* = 18)]. That is, vegetation + grassy zone, vegetation zone, open sand, and grassy areas had obvious differences in term of hawksbill preferences of nesting across two years of study. In addition, hawksbills were observed to nest above the high tide line, and the most preferred distances was from 11.0 m to 20.9 m, 49.2%. The overall mean distance was 15.6 ± 10.1 m (median = 14.6 m, *N* = 295) and ranged from 2 to 90.0 m above the high tide line.

## DISCUSSIONS

The updated statistics of hawksbill nesting are between 300 nests–600 nests per year between 2015–2019. These results show good conservation efforts by the DoF and Padang Kemunting Turtle Conservation Centre as the density of nesting has increased and remained stable since it was last documented in 2013–2014. In previous researches, a yearly number of nests was estimated to be approximately > 350 nesting as documented in 1992–1993 (Mortimer *et al*. 1993), and between 400–500 nesting documented in 2013–2014 (Sarahaizad *et al*. 2018). This sign of recovery is the result of effective management by the DoF, including deploying sufficient patrollers on each beach to control poaching and mitigate human disturbances, especially at beaches with active beachfront activities. Extended beach patrol hours have had a positive impact and contributed to the reduction of egg poaching. The increase in hawksbill turtle numbers in Melaka after 30 years is considered a success of long-term conservation efforts. This is due to hawksbill population in Brazil, which share the same tropical weather with Malaysia, shows apparent increasing trend in nesting seems to reflect a range of conservation measures implemented over the past 25 years (Marcovaldi *et al*. 2007). No green turtle landing was recorded within five years survey, and the similar results was reported in 1991 (Mortimer *et al*. 1993) and 2013–2014 (Sarahaizad *et al*. 2018).

The findings in this study disclose there was a significant effect of nesting density with air temperature and rainfall, which shows the environmental influence on nesting behaviours in terrestrial habitats. Multiple environmental cues may be important to the female sea turtle when deciding where to nest, and a combination of cues may help to explain more variation in nesting patterns at different beaches, than a single environmental variable (Pike 2008; Antworth *et al*. 2006). The peak nesting period in Melaka was observed to occur between June to August. This is because the wind and rainfall during this period (from late May or early June to the end of September) are milder compared to the stronger winds experienced during the northeast monsoon season, which occurs from November to March (Malaysian Meteorological Department 2021). In certain cases, hawksbill turtles have demonstrated an ability to adapt to elevated temperatures. The finding by dos Santos Oliveira *et al*. (2020) suggests that hawksbill populations are actively responding to warming environmental conditions by shifting the peak of nesting season, and the number of nests remained stable. Numerous environmental conditions may also influence the behaviour when a female turtle selects a nesting site (Halls & Randall 2018).

In Melaka, a peak was seen between June and August and showed a year-round nesting. Year-round nesting is considered as common for hawksbill, as been observed in tropical regions countries such as the Dominican Republic (Revuelta *et al*. 2012; 2013), the Caribbean (Kamel & Delcroix 2009; Beggs *et al*. 2007), and Indian Oceans (i.e., Solomon Island [Hamilton *et al*. 2015] and Seychelles [Mortimer *et al*. 2011]).

Except in Gulf regions, the pattern of nesting observed occurred starting from April until the early weeks of July in every year. This seasonal pattern was recorded occurred in Oman (Mendonça *et al*. 2001), Qatar (Chatting *et al*. 2018), Saudi (Al-Merghani *et al*. 2000) and UAE (Al-Ameri *et al*. 2022) due to environmental factors, and climate change also can cause to habitat alterations (Aini Hasanah *et al*. 2014; Patel *et al*. 2016). Most interestingly, even though hawksbill in the Gulf region only nested at a certain period, the number of nests collected in Qatar (> 300, Chatting *et al*. 2018) and Oman (> 500, Mendonça *et al*. 2001) was almost the same or just slightly lower than the hawksbill population in Melaka that nested throughout the year.

In addition, the trend for hawksbill season starts to increase from June onwards in certain regions globally. Similar peaks for nesting were also observed in Barbados, June–August (Beggs *et al*. 2007), Dominican Republic, June–November (Revuelta *et al*. 2012), Solomon Island, May—July (Hamilton *et al*. 2015), and Australia, July–October (Limpus *et al*. 2000). Different behaviour performed by the hawksbill in the western Indian Ocean where nesting occurs year-round but peaks was in December to January (Phillips *et al*. 2014), and this is similar to nesting behaviour in northern Brazil (Vieita & Godfrey 1999).

A maximum of 110 nests were recorded at Padang Kemunting during the initial observation period in 2013–2014 (Sarahaizad *et al*. 2018), increasing to a peak of 188 nests in 2017. This upward trend over the five years reflects improved nesting activity, likely influenced by enhanced management efforts between local authorities and the community. Padang Kemunting is a tourist attraction beach and one of the beaches that is dominated by beach-front resort activities. Most of the resort’s owners are the local people and DoF are hiring them as licensed egg collectors. As a result, the residents also cooperating in collecting the eggs and selling those eggs to the DoF. Poaching still occurs in Melaka; however, through this cooperative approach, the previously uncontrollable poaching rate has been reduced. This improvement is attributed to the positive relationship between authorities and local communities and villager. The poaching rate decreased by approximately 27.9% in 2018 compared to the previous year, 2017 (Table 4). Fully poached nests (those with no eggs remaining) also showed a 33.8% decline over the one-year observation period. Patrollers monitored the entire length of the beach, from beginning to end, although some nests may have been overlooked, particularly at the start of the patrol area. Since the beaches in Melaka are not gazetted as turtle sanctuaries, the DoF relies on cooperation from villagers to prevent nest poaching. Any cases of partially (poached nest with remaining eggs) or fully poached nests are reported to the police, as only licensed egg collectors are permitted to collect turtle eggs. This regulation is enforced under the Fisheries Act 1985 (Amendment 1993) and the Fisheries Methods (Turtle and Eggs) 1989 under the jurisdiction of the State Government. These laws were enacted to ensure the responsible handling of turtle eggs, with a key provision being the licensing of egg collectors. All collected eggs are then incubated at the hatchery in Padang Kemunting. In every year, Padang Kemunting accepts high human density due to near-shore and ocean recreation activity (i.e., boating, fishing, beach camping), especially during the holiday season. By placing sufficient patrollers and interaction between the authority government-local peoples, the DoF team has managed to minimise night-time human disturbances especially during the important festival season occurred in June–July.

More interestingly, an increase in nesting density has also been observed at nesting beaches with high human activity (Table 1). For example, in Kem Terendak, Tanjung Dahan and Meriam Patah. For example, from 57 nests collected in 2014 at Kem Terendak, the number had increase to 50 nests–130 nests collected between 2015–2019. Similar observation occurred at Tanjung Dahan, from 30–50 nests to 10–100 nests, and Meriam Patah, where nests increased from between 43–48 nests to 30–70 nests. Beaches also provide valuable ecological services in the form of ingenious protection for adjacent habitats and crucial nesting habitats for marine turtles (Hendry 1993). Therefore, hawksbill turtles appear to force themselves to nest under extreme conditions and adapt to their surroundings, likely as a response to long-term anthropogenic disturbances in their natural habitats. It was observed hawksbill performed a rapid process of digging nests, probably cause turtle able to overcome the anthropogenic disturbances and able to lay eggs on the beach with high human activity (Ferreira & Martins 2013).

A stable decline was observed in Tanjung Serai, Pasir Gembur, Tanjung Bidara, Balik Batu and Teluk Belanga as these beaches recorded 29–60, 42–36, 2–10, 23–53 and 7–26 nests in 2013–2014, and the result was almost the same number of nests collected from 2015–2019 (Table 1). Certain beaches show a decrease in density (Pulau Upeh and Sungai Udang). Pulau Upeh shows a drastic decline due to its proximity to the nearest land reclamation site causing to rapid coastal erosion and degrades the once-pristine beach (New Straits Times 2018). Since the reclamation, the number of nests in Pulau Upeh has decline year by year. The reduction of nests at Sungai Udang are also believed due to impact on coastal development. Nowadays, cooperation between the tourism and conservation sectors is essential to prevent a drastic decline in the nesting population, especially in areas affected by active land reclamation and beachfront tourism activities.

In Melaka, hawksbill turtles prefer to nest at night. A similar pattern has been observed in the Caribbean, where nesting primarily occurs during nocturnal hours (21:00 to 03:00) (Walker & Gibson 2015). This region shares a similar tropical climate with Malaysia. In Melaka, nesting typically occurs between 20:00 and 02:00, further confirming the species’ preference for nocturnal nesting behaviour. Turtle landing at high tide decreases the distance the turtle must crawl to reach the nesting area, thermal stress and desiccation (Burger & Montevecchi 1975). Turtles nesting usually occur at midnight and a majority of turtles emerges during high tide (Burger & Montevecchi 1975), as it decreases the time and energy for female turtles to land on the beach. In addition, beaching at high tide decreases the distance the turtle must crawl to reach the nesting area, thermal stress, and desiccation, and increases the turtle’s chance of nesting above high tide (Burger & Montevecchi 1975). Small occurrence of diurnal nesting was considered as common for hawksbill turtle in Seychelles with 32 occurrences reported within eight years period (Beggs *et al*. 2007). In Melaka, they are approximately 40 diurnal nestings recorded in between 06:00–08:00 h within two years study period.

They are solitary nesters, hawksbill often nesting at the far end of beaches close by vegetative berms (Murphy *et al*. 2018; Nasiri *et al*. 2022). Vegetation is an important factor in hawksbill nest site selection in this study and other nesting beaches (Ficetola 2007; Kamel & Delcroix 2009; Zare *et al*. 2012; Tomillo *et al*. 2014). In this study, we found hawksbill prefers to nest within the vegetation zone. A general explanation for this is that nest sites with vegetation zone are often less compacted than those without the vegetation (Horrocks & Scott 1991). The existence of woody vegetation at a certain distance from tide lines may provide optimal humidity and temperature (Foley *et al*. 2006; Zare *et al*. 2012). Higher sand temperature generally promotes marine turtle nesting (Sato *et al*. 1998; Chatting *et al*. 2018) suggested between 25°C to 34°C (Bustard & Greenham 1968), and root from woody vegetation provides stability for the nest avoiding nest rupture. In addition, vegetation may limit the erosion from increased sea levels and storm frequency. Therefore, vegetation is considered a critical component of the hawksbill nesting habitat. Hawksbill’s preferred distance was from 11.0 m to 20.9 m and distance ranged from 2.0 m to 90.0 m above the high tide line. Different results were found in the Persian Gulf; most hawksbills nested at a distance of ≤ 15 m from the high tide line with 71.8% observations, and generally ranged from 1 m to 3 m (83.9%) above sea level (Nasiri *et al*. 2022). Beach width and ecology conditions do impact the distance of nesting. For example, the female turtle tends to nest at a certain distance above the high tide line probably related to optimal sand temperature and humidity (López-Castro *et al*. 2004). Optimal temperature is important to allow good nest ventilation (Ackerman 1980), and less humidity results in a less binding effect during nest construction.

## CONCLUSION

This article provides an updated overview of the nesting status, seasonality, and breeding biology of hawksbill turtles in Melaka from 2015 to 2019. These findings reflect the outcomes of ongoing conservation efforts by the Department of Fisheries (DoF) aimed at protecting the species from the impacts of human activities. The protection and monitoring of hawksbill turtles are especially critical in Melaka, where they face increasing threats from coastal development and land reclamation projects. Therefore, this article highlights the current status and challenges facing the hawksbill population in the region.

## Figures and Tables

**TABLE 1:** Coordinates of the beaches, nesting beaches and number of nests.

No.	Coordinates		Beaches	Nesting density					Sum of nesting
	
	Latitude	Longitude		2015	2016	2017	2018	2019	
1	2.3161	102.0704	Padang Kemunting	85 (20.3)	119 (31.1)	188 (32.8)	157 (26.2)	117 (32.3)	666
2	2.2852	102.1062	Kem Terendak	54 (12.9)	66 (17.2)	93 (16.2)	125 (20.8)	128 (23.4)	466
3	2.3659	102.0318	Tanjung Dahan	66 (15.8)	65 (17.0)	19 (3.3)	86 (14.3)	94 (17.2)	330
4	2.3699	101.9832	Meriam Patah	47 (11.2)	30 (7.8)	62 (10.8)	37 (6.1)	49 (8.9)	225
5	2.3747	101.9788	Tanjung Serai	51 (12.2)	24 (6.3)	62 (10.8)	44 (7.3)	32 (5.8)	213
6	2.3008	102.0780	Pasir Gembur	21 (5.0)	15 (3.9)	28 (4.9)	29 (4.8)	38 (6.9)	131
7	2.3511	102.0333	Teluk Belanga	27 (6.5)	21 (5.5)	19 (3.3)	45 (7.5)	6 (1.1)	118
8	2.2964	102.0786	Balik Batu	17 (4.1)	15 (3.9)	28 (4.9)	16 (2.7)	39 (7.1)	115
9	2.1936	102.2033	Pulau Upeh	15 (3.6)	13 (3.4)	36 (6.3)	20 (3.3)	16 (2.9)	100
10	2.3097	102.0724	Pengkalan Balak	11 (2.6)	3 (0.8)	7 (1.2)	8 (1.3)	14 (2.6)	43
11	2.1796	102.2743	Kampung Teluk	8 (1.9)	2 (0.5)	26 (4.5)	5 (0.8)	0 (0.0)	41
12	2.3381	102.0602	Sungai Tuang	2 (0.5)	0 (0.0)	1 (0.2)	14 (2.3)	12 (2.2)	29
13	2.3808	101.9767	Kuala Linggi	5 (1.2)	7 (1.8)	4 (0.7)	4 (0.7)	0 (0.0)	20
14	2.3412	102.0500	Teluk Gong	2 (0.5)	0 (0.0)	0 (0.0)	9 (1.5)	2 (0.4)	13
15	2.2929	102.0875	Tanjung Bidara	2 (0.5)	2 (0.5)	0 (0.0)	0 (0.0)	0 (0.0)	4
16	2.2739	102.1011	Sungai Udang	3 (0.7)	0 (0.0)	0 (0.0)	0 (0.0)	0 (0.0)	3
17	2.3291	102.0670	Sungai Kertah	1 (0.2)	1 (0.3)	0 (0.0)	0 (0.0)	0 (0.0)	2
18	2.368	102.0068	Kampung Tengah	1 (0.2)	0 (0.0)	0 (0.0)	1 (0.2)	0 (0.0)	2
19	2.2474	102.1370	Puteri	0 (0.0)	0 (0.0)	0 (0.0)	0 (0.0)	1 (0.2)	1
20	2.2263	102.1495	Tanjung Kling	0 (0.0)	0 (0.0)	0 (0.0)	0 (0.0)	0 (0.0)	0
21	2.2181	102.1768	Kelebang	0 (0.0)	0 (0.0)	0 (0.0)	0 (0.0)	0 (0.0)	0

Sum				418	383	573	600	548	2,522

**TABLE 2:** Sum of eggs and survival hatchlings over five-years under the management of Padang Kemunting Turtle Conservation Centre, Melaka, Malaysia.

Year	Total nests	Total eggs	Total hatchlings released	Hatching success (%)
2015	418	47,248	26,842	56.811
2016	383	42,248	16,067	38.030
2017	573	64,398	38,932	60.455
2018	600	71,044	43,586	61.351
2019	548	66,695	36,759	55.115

Total	2,522	291,633	162,186	55.61

**TABLE 3:** Poached nest records in 2017 and 2018 in Melaka, Malaysia. A partially poached nest refers to a nest where some eggs remain after poaching. In contrast, a fully poached nest refers to a nest where no eggs are left.

No.	Nesting beaches	2017			2018		

		Whole nest that was poached	Nest that was partially poached	Overall sum in 2017	Whole nest that was poached	Nest that was partially poached	Overall sum in 2018
1	Padang Kemunting	23	3	26	10	7	13
2	Pengkalan Balak	10	2	12	2	1	4
3	Kem Terendak	3	0	3	4	2	4
4	Balik Batu	13	19	32	0	2	19
5	Teluk Belanga	0	2	2	1	1	3
6	Tanjung Dahan	9	1	10	21	1	22
7	Meriam Patah	2	0	2	6	0	6
8	Tanjung Serai	13	0	13	4	0	4
9	Pulau Upeh	4	0	4	0	0	0
10	Teluk Gong	0	0	0	0	0	0

	Total	77	27	104	48	14	75

**TABLE 4:** Linear regression analysis between nest and mean clutch size with climate factors.

	Environmental factors			

	Temperature (°C)	Humidity (%)	Precipitation (mm)	Rainfall amount (mm)
Sum of nest	Nest = −3.64 + 14.6; Temperature, *P <* 0.05 (sig.)	*P* > 0.05	*P* > 0.05	Nest = 20.0 + 0.12; Rainfall, *P* < 0.05 (sig.)
Mean clutch size	Mean clutch size = 277 – 5.7; Temperature, *P* < 0.05 (sig.)	*P* > 0.05	*P* > 0.05	*P* > 0.05
